# The Utilisation of Isolation Hospitals as Sanatoria

**Published:** 1912-05-04

**Authors:** 


### SANATORIUM CONSTRUCTION.

### The Utilisation of Isolation Hospitals as Sanatoria.

Occam formulated a logical principle of great subsequent use when he forbade us to impute an effect to some abstruse cause while simpler explanations remained undisproved. The experience of the last twenty years with regard to tuberculosis justifies this principle abundantly. Measures like residence abroad, or gentle horse exercise, or sea voyages, which from early times had been found to benefit the consumptive, are now deemed to owe their therapeutic efficacy not to mysterious and undefinable intrinsic virtues, but to the fact that they all involve prolonged exposure to the open air. The establishment of sanatoria in many different localities, at different altitudes, and with various surroundings (consult, for instance, p. 556 of the late Dr. Bulstrode's well-known Report) showed that responsible people were willing to venture on this view, and the result—a pretty even level of success, other things being equal—proved that this view was the correct one. At least two of our large modern sanatoria are placed, as an actual matter of fact demonstrable with a No. 3 garden-spade, on stiff clay soil, and they do just about as well as the others. Still, when a choice is available sanatorium promoters naturally go for pine trees and agreeable scenery and gentle ascents. That is right enough; for these amenities, to use a word of Aristotle's, are certainly choiceworthy. But it should now be fully recognised that they are also altogether subordinate in importance to that greatly-to-be-considered aspect of the tuberculosis problem, namely, the economic one.

We see that Lancashire hospital authorities have realised as much. It appears that at a quarterly meeting of the Fylde, Preston, and Garstang Joint Hospital Board the clerk stated he had received a communication from the Local Government Board declining to grant power to use the Elswick Smallpox Hospital for cases of consumption, as their inspector considered the site unsuitable. Some hitch seems to have occurred, since the Local Government authorities are stated to have themselves suggested the advisability of applying for the order. This hitch, it was surmised, arose from the fact that on the day of the inspector's visit an abnormal rain- and snow-fall had caused the building, which had never been occupied, and the site, to appear at their worst. Moreover, there was plenty of medical evidence in favour of the accommodation and surroundings as suitable for consumptives. Ultimately a deputation of four was appointed to interview the Local Government Board and discuss the question of how the building could be used, since there had been in the past and there seemed likely to be in the future no cases of smallpox.

We understand that the Local Government Board have not yet consented to receive this deputation, the reason probably being that the site is considered to be water-logged, and, therefore, wholly unsuitable for the purposes of a sanatorium. As a general thing smallpox hospitals are rather well suited, with a little alteration, for conversion into sanatoria, since they have grounds round them and the initial air-space is on a liberal scale. Hence the process of transformation should not run into much money. It is true that Kuthy is quoted by Dr. Walters as giving the cost of alteration as £128 per bed, but then this particular instance referred to an ordinary house in Berlin. Again, if the place had never been used, it is likely enough stormwater channels had not been provided for very carefully, and such a wet spell as has occurred recently would make the locality look very uninviting. More to be considered than any of these points, except the first, is the fair size of the building. If the cost of £22,000 is correctly stated, it should be able to house a good number. This in practice is a very great matter, small numbers not being able to support a proper administration. The patients then get into a lax way, presently some of them misbehave out of doors, and the institution becomes a nuisance to the neighbourhood, to the lasting discredit of sanatoria. But with a building of the size we are left to infer, and with the addition of a pavilion or two as close to the main block as possible, a well working unit under male resident management could be set up with very little delay and comparatively small expenditure. In dealing with a disease as common amongst the poor as consumption, it is most especially imperative to cut the coat according to one's cloth.

